# Digital technology and human development: A charter for nature conservation

**DOI:** 10.1007/s13280-015-0703-3

**Published:** 2015-10-27

**Authors:** Georgina Maffey, Hilary Homans, Ken Banks, Koen Arts

**Affiliations:** dot.rural, University of Aberdeen, MacRobert Building, King’s College, Aberdeen, AB24 5UA Scotland; Centre for Sustainable International Development, University of Aberdeen, MacRobert Building, Aberdeen, AB24 5UA Scotland; Cambridge, UK; Forest and Nature Conservation Policy Group, Wageningen University, Droevendaalsesteeg 3, 6700 AA Wageningen, The Netherlands; Centro de Pesquisa do Pantanal, Universidade Federal de Mato Grosso, Cuiabá, CEP 78.068-360 Brazil

**Keywords:** Charter of best practice, Nature conservation, Mobile phone, Developing world, Information Age, Innovation

## Abstract

The application of digital technology in conservation holds much potential for advancing the understanding of, and facilitating interaction with, the natural world. In other sectors, digital technology has long been used to engage communities and share information. Human development—which holds parallels with the nature conservation sector—has seen a proliferation of innovation in technological development. Throughout this Perspective, we consider what nature conservation can learn from the introduction of digital technology in human development. From this, we derive a charter to be used before and throughout project development, in order to help reduce replication and failure of digital innovation in nature conservation projects. We argue that the proposed charter will promote collaboration with the development of digital tools and ensure that nature conservation projects progress appropriately with the development of new digital technologies.

## Introduction

The current era in the history of humankind has been described as ‘the Information Age,’ a period characterized by the increasing use of digital technology to mediate access to, and management of, information (Mason 1986; Castells 2010). Like many other societal domains, the environmental sciences have embraced digital technology to manage information and enhance analytical power (Stafford et al. 1994, p. 3). The establishment of sub-disciplines such as ecological modeling and bioinformatics, as well as the embedded use of digital technology within others (e.g., Geographic Information Systems—GIS), is testament to this.

Discussion on the use of digital technology in the context of nature conservation (hereafter conservation) in its broadest sense^1^ is less developed (Arts et al. 2015a). Those academic studies that have begun to consider the use of digital technology in conservation have cited factors such as cost (Graham et al. 2012), durability (Stevens et al. 2013), and data integration (Teacher et al. 2013) as key challenges in this area. Yet, the same studies also emphasize the potential that digital technology holds to improve data collection in the field to share information and to empower local communities involved in conservation.

Domains such as health, education, and human development have embraced the notion of empowerment, and continue to explore the use of digital technology as a facilitator of attitudinal or behavioral change—e.g., investment in telemedicine (Rosser et al. 2009). Consequently, such domains can offer insight into how digital technology might best be used in conservation; in this sense, conservationists may, for instance, be able to ‘leap frog’ the development of inappropriate tools.

The aim of this Perspective is to explore the potential lessons that conservationists can learn from other domains on using digital technology as a tool to meet conservation goals. Due to parallels between the conservation and human development domains (Adams et al. 2004; Büscher and Dressler 2012), we focus primarily on examples from human development, a domain that has capacity-building characteristics (i.e., the ability of individuals and organizations to perform functions, solve problems, and set and achieve objectives—United Nations Economic and Social Council 2006). We conclude with a proposed charter of best practice for the application of the lessons cited and the constructive future use of digital technology in conservation.

## Background

### Three dimensions

This Perspective revolves around three dimensions: digital technology, human development, and conservation. While there are parallels between human development and conservation, it should be considered that different key drivers are behind changes in each of the three dimensions. Human development is influenced by cultural, economic, environmental, political, and social factors that affect people (Malik 2014). Thus innovation in human development tends to focus on capacity building (United Nations Economic and Social Council 2006) either to mitigate (potential) threats or to improve the status quo. Because of this trend, we consider human development as being problem driven.

The development of digital technology is, on the whole, market driven due to influences from the commercial sector—e.g., competitive innovation, as companies file for patents to protect their technological developments. However, at the interface of human and technological development innovation often occurs through non-profit organizations and is problem orientated in its design. Such innovations tend to arise from an open-source approach, which can result in further innovation in technology use by others. Designing digital technology to address problems in this way can also be influenced by the availability of funding, which in turn may result in the replication of projects that do not fully address the actual problem (cf. Araral Jr 2005). Thus, while competition does exist in the non-profit sector, it has a fundamentally different character to that in the commercial sector (Lall 1993).

Conservation can be thought of as mission driven or concern driven (Soulé 1985; Meine et al. 2006; Mace 2014) with desires to protect landscapes and species that are (potentially) threatened by anthropogenic factors. Such motivations to conserve hold clear similarities to those that underpin problem-driven human development, and the allocation of funding to support conservation projects is strongly influenced by social pressure and public policy (Czech et al. 1998; Ferraro and Pattanayak 2006). However, it is often more difficult to see the results of conservation efforts within the same timeframes as those of human development projects. This may in part be due to the fact that on a day-to-day basis, conservation issues do not always have the same urgency for individuals as other domains and facets of modern-day life (Jepson and Canney 2003). Yet as such, technology, which is increasingly integrated into modern-day life, may provide an opportunity to facilitate a connection between conservation and other domains.

### Digital connectors

There have arguably been two key developments that have disproportionately influenced individuals’ behaviors in the Information Age: the Internet and the mobile phone (Schwanen and Kwan 2008). The Internet acts as a mass connector, shaping modern society in myriad ways (Castells 2010), with implications for security, privacy, politics (Shah et al. 2005), and social justice (Jones 1997). Yet, access to the Internet is not yet a global privilege. While individuals and institutions may generally appear to be better digitally connected, such connection can vary considerably both across and between communities^2^ (Kvasny et al. 2006; Newman et al. 2010).

According to the International Telecommunication Union (ITU), in 2014 approximately 40% of the global population was using the Internet (ITU 2015a). In developed^3^ countries, 78 % of the population were Internet users, but in developing countries this was just 32 % of the population. The figures on Internet use contrast starkly with those on mobile cellular subscriptions. Mobile cellular subscriptions have more than trebled globally since 2005, and it was estimated that at the end of 2014 subscriptions numbered almost 7 billion, of which 78 % (5.4 billion) were held in developing countries. It is because of the continued growth of mobile phone use in the developing world, and the majority of the examples discussed in this paper are centered on mobile, rather than Internet, applications.

At present, access to, and use of, mobile phones in many developing countries is largely an urban phenomenon. This, in combination with cultural factors, can result in ‘usage gaps’ (Van Dijk and Hacker 2003) and leave some groups without access, e.g., women, persons with disabilities, people living in poverty, and the elderly (Kvasny et al. 2006; ITU 2015b). Despite these tendencies, the increasing availability of cheap handsets (e.g., Google’s Android One—an affordable smartphone released in India^4^), pre-paid price plans, and greater network coverage (Donner 2007) have provided opportunities for individuals and communities in developing countries to connect locally, regionally, nationally, and globally (Gumpert and Drucker 2007, p. 10). These factors, in combination with resource scarcity and less-developed cyber infrastructures, have resulted in mobile sector social innovation in developing countries often being cultivated differently to that in developed countries (Donner 2007). The so-called ‘leap frog’ hypothesis (Howard 2007) encapsulates the idea that developing countries can capitalize on the lessons learnt by other countries. Developing countries can thus effectively fast track their path toward becoming an Information Society.

In this Perspective, we illustrate potential lessons for conservation with examples, many of which refer to projects where digital technology has been used in an innovative way to positively influence human development. Consequently, the use of examples that have been successful in their implementation means that there is a positive bias to the projects cited, as is often seen in innovation literature (cf. Rogers 2003; Maffey 2014). Digital technology can equally be used as a tool to negatively influence human development (cf. Weeramantry 1993) and conservation (Büscher 2013; Sandbrook et al. 2014). However, throughout this Perspective we explore the characteristics of the successful implementation and operation of technology in human development projects. In doing so, we hope that the proposed charter, which stems from the lessons learnt through this exploration, can be used as a frame to effectively ‘leap frog’ the use of technology in conservation projects.

## Lessons for conservation

### Updated and outdated

Market drivers influence technological development in the commercial sector often with small and frequent incremental updates of software and hardware used to encourage continued consumer investment in a product (Hills and Sarin 2003; see Box 1). As new pieces of hardware and software are introduced, opportunities can arise for emerging initiatives, as was seen with the rise of M-PESA—a mobile banking scheme—which originated in Kenya and is now being used in many countries across sub-Saharan Africa (Omwansa 2009). However, it can also present challenges as some charitable organizations struggle to maintain products consistently alongside the open market.

**Box 1:** Case Study 1: Personal Digital Assistants—out of date before it’s built

While working on infectious diseases with the Centers for Disease Control and Prevention (CDC), Joel Selanikio, a medical doctor, found that the collation of public health data in developing countries was inherently problematic (Banks 2013). The process of dissemination, collation, and analysis of paper-based public health data collection forms could take years—with instances of data never actually being entered into a computer
In 1998, Selanikio identified an opportunity to change the way that data collection occurred and piloted a nutrition survey with US Army nutritionists and Burmese refugees in a Thai refugee camp, using software on Personal Digital Assistants (PDAs—mobile devices that allow storage and management of information). Despite having some success in collecting data and publishing (Selanikio et al. 2002) on the use of the PDA software, there was little adoption of the system
Selanikio identified difficulties with the complexity of establishing and using the digital forms. Together with Rose Donna from the American Red Cross, he developed a second simpler data collection system, where data could be collected on a PDA and then collated and analyzed on a computer
In 2009, Selanikio replicated the system but now as a web application—inspired by the rapid global growth of programs such as Hotmail and Google. Soon after this, Selanikio was able to run the system on a mobile phone (rather than a PDA) to compliment the web application. In doing so, individuals were able to access the platform much more cheaply and simply, and across multiple operators. As Selanikio’s system began to grow, the PDA market collapsed—if Selanikio had not continued to pursue cheaper and more accessible technology, the system he had developed would have disappeared with the PDA market collapse
Selanikio’s product development highlights the importance of maintaining an awareness of the technological climate, and ensuring that a project is not focused on a single platform that stands to be influenced by short-term changes or technological advances

For conservation projects, similar problems arise in how to address evolving digital technology and its use. In conservation, digital technology is not always developed with communities, but instead introduced as a tool to engage with communities (e.g., citizen science). Digital tools require significant and continued investment in their use, with a continual need for updates of software and hardware. For small organizations, the burden of maintaining such a tool can be large, and it raises the question as to whether organizations should invest in technologically capable individuals interested in conservation or train conservation-focused individuals in technological development.

Conservation projects often deal with timelines that far exceed those of a single human generation. This comes with issues on an individual level, such as Shifting Baseline Syndrome (Kahn 2011, p. 165), in that individuals are designing solutions to address what *they* currently recognize to be pertinent environmental issues. The Shifting Baseline Syndrome may be amplified by a mind-set in which digital technology is applied as a short-term fix to conservation challenges. In this sense, there is a need to ensure that conservation is not strongly influenced by market forces that promote the latest ‘must-have’ technological innovation (cf. Heinonen et al. 2001). As is clear from Case Study 1, it is appropriate to consider existing and persisting platforms (from other domains) and maintain an awareness of the fact that technological applications will become outdated.

### Reinventing the wheel

One of the main drivers of the development of digital technology is innovation, the process of generating a new idea, product, or method. Innovation in digital technology is due, in part, to the existence of an open and free commercial consumer market, in which competitive digital development occurs. In instances where human development and the development of technology overlap, the idea of reluctant innovation has been proposed. In reluctant innovation, an individual is so strongly (negatively) affected by an issue or problem that they are compelled to find a solution. For example, there are numerous innovations from organizations that are designed to improve energy provision in rural areas of sub-Saharan Africa (cf. Karekezi and Kithyoma 2002; Krebs et al. 2010). However, a reluctant innovation would develop out of necessity, such as in the case of William Kamkwamba who built windmills to provide his own village with electricity.^5^ Across sustainable human development, reluctant innovation has resulted in digital platforms to address issues in health, education, and energy provision (Banks 2013; Box 2), and it may be that similar examples of reluctant innovation begin to emerge with increasing (negative) pressures on the natural environment.

**Box 2:** Case Study 2: FrontlineSMS—innovation in mobile technology

FrontlineSMS (http://www.frontlinesms.com/) was created in 2005 to enable effective communication channels for communities in the developing world. For most of the developed world, it was becoming commonplace to rely on the power of the Internet for several aspects of daily life. But across much of sub-Saharan Africa, with less than 10 % of the population online in 2005, information access was scarce. Ken Banks was early to recognize the potential of mobile phones, specifically text messaging, to disseminate information, organize aid, and reconnect communities in times of crisis. But individual phones could not easily broadcast to large groups. So Banks pioneered a method for turning a laptop or desktop computer into an offline hub for two-way text messaging, independent of a continuous Internet connection
Initially Banks’ concept was intended for use in ecologically threatened regions of sub-Saharan Africa. But the free, open-source, and user-centered design of the software that leverages the simplicity and familiarity of texting has allowed citizens and grassroots organizers to adapt it for other purposes. FrontlineSMS has since become an engine for bottom-up social change, from promoting literacy in Niger, and assisting family farmers in Laos, to training rural medics in Ecuador. It has enabled group communication in situations of civil war, political upheaval, or natural disaster. Moreover, by working with existing tools and infrastructure FrontlineSMS has helped to increase information access across and between communities in a way that minimizes duplication of similar tools

Regardless of how innovation is stimulated, there is a danger that, in the proliferation of ideas, products, or methods, (pilot) projects are repeated. When there is a lack of collaboration across organizations, multiple solutions to a single problem can be created (Dichter 2003). In developing countries, a lack of collaboration can mean that many innovations do not fulfill their original intentions, especially when insufficient attention has been paid to local context (Seyfang and Smith 2007). There is a danger that, if the same occurs in conservation, innovation will place an emphasis on novelty or ‘fads’ rather than progress (Redford et al. 2013). It is likely that many conservation issues can be addressed (in part) by working in collaboration with local communities (Lewis 2012) and adapting existing tools. The use of technology should be continually questioned to ensure that it is relevant, serves wider conservation goals, and does not reinvent the wheel.

### Taking a bottom-up approach

The introduction of a digital solution *into*, as opposed to an initiative that comes from *within*, a community can be problematic. Initiatives that do not incorporate a bottom-up approach to development may lack knowledge of, or misunderstand, the local context. Access to digital technology can contribute to digital divides and accentuate existing societal inequalities (Thompson 2004), as “historically, technologies have been used by those in power to retain their positions of power” (Unwin 2012). Such power dynamics can affect if and how technology is adopted (Arts et al. 2013; Maffey 2014). However, when communities self-run projects and deploy digital technologies themselves (Thioune 2003; Donner 2007), the associated level of local ownership can result in the proliferation of secondary opportunities and entrepreneurship (see Box 3).

**Box 3:** Case Study 3: Wildlife-CoMMS—development from the ground up

The Wildlife-Conservancy Management Monitoring System (Wildlife-CoMMS) is a basic system for monitoring trends in wildlife ecology, for example, regarding changes in species abundance or levels of poaching. It is used by community conservancies in northern Kenya; “Community conservancies are community owned organisations, which aim to improve biodiversity conservation and livelihoods of local people over a defined area of land traditionally owned, or used, by the constituent community” (Northern Rangelands Trust 2015; http://www.nrt-kenya-comms.org/)
Wildlife-CoMMS was designed by communities and comprised a series of guides, which demonstrate how to collect and collate data on wildlife ecology, and a digital database that enables conservancies to manage and visualize their data through mapping. The Northern Rangelands Trust (NRT)—the umbrella organization for community conservancies—developed the guides through trial and implementation over a seven-year period. The design of the guides was initially piloted in one conservancy, and they are now used in 17 NRT conservancies by over 300 community rangers
The success of the guides and the digital database has largely been due to the continued feedback from conservancy managers, wardens, and (other) local stakeholders. This has allowed the creation of a system that is appropriate in the context of community conservancies. The focus on involvement with local communities has resulted in a tool that “empowers those who live on the land to better understand and protect their natural resources” (Michelmore-Root 2014, pers. comm.)
The Kenyan government has now endorsed this tool for use outside of designated protected areas in traditional pastoralist areas where humans, wildlife (including key endangered species), and livestock co-exist. The success of the system has led to its application in areas outside Africa, such as on Fraser Island, Australia

**Fig. 1:**
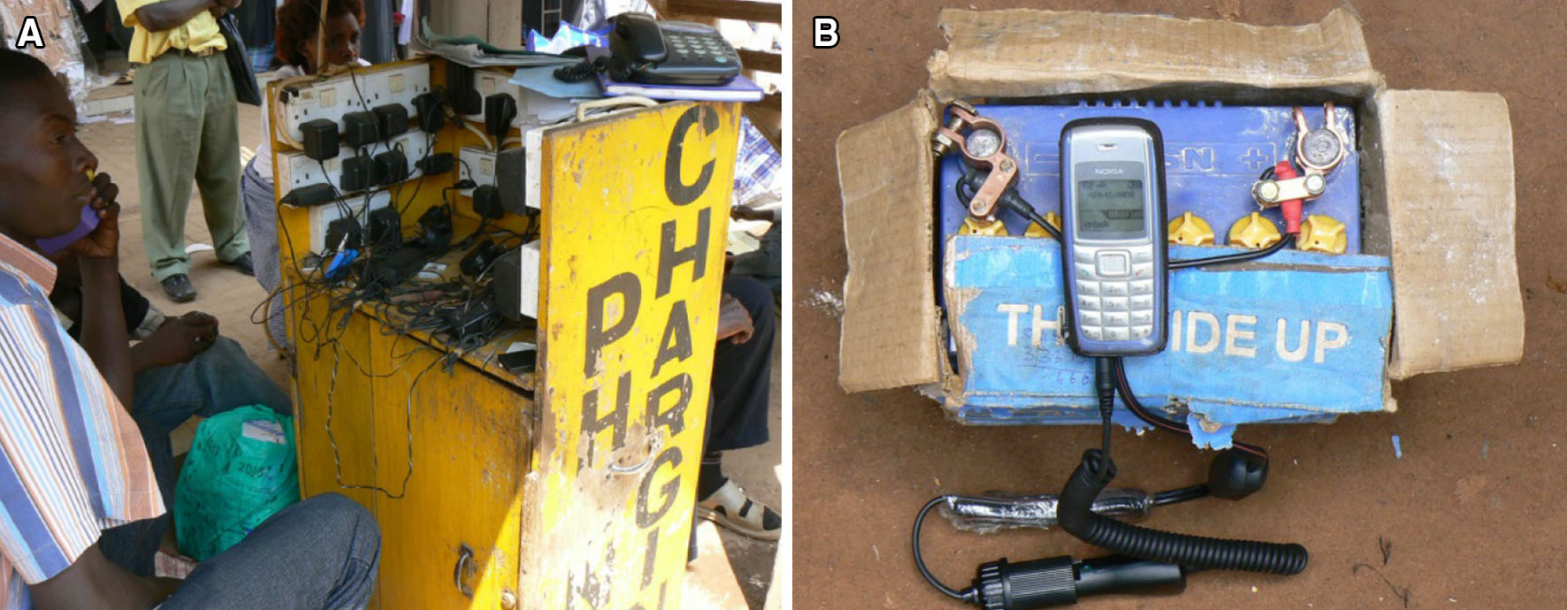
**a** Mobile phone charging station, Uganda; **b** car battery village phone charger, Uganda

The use of digital technologies by local communities is often reliant on a level of national commitment in the development of plans and investment in modern infrastructure (Webersik and Wilson 2009). The introduction of digital technology on a wide geographical scale is usually centrally administered by, e.g., public bodies, and can be biased toward urban investment. However, where this investment is lacking (e.g., in rural areas; Wyche and Murphy 2013), small- and medium-scale enterprises often address subsequent challenges. For example, in many developing countries the absence, or erratic supply, of electricity presents difficulties for the regular charging of mobile phones, and different entrepreneurial solutions have arisen, such as the use of mobile stations, or car batteries, as phone charging points (Lindsay 2015; Fig. 1).

In the ‘Diffusion of Innovations’ model, the importance of individuals, or groups, who act as champions within the community is emphasized (Henrich 2001; Rogers 2003). As the model suggests, the success of a project or service is often reliant on early and continued engagement with or ownership and communication of the development by the local community. Implementing, or attempting to implement, technology without buy-in and input from local communities is likely to result in failure (Rogers 2003). Ideally, the community itself should be the instigator of the intervention or development. However, lack of access to information, education, and knowledge sharing can limit such instigation, and it is in those cases that organizations can play a vital role in helping communities to determine and influence the benefits that a technology may offer.

The importance of local context is something that many conservation organizations, particularly those working in remote or rural areas, are already acutely aware of. However, the importance of local context becomes particularly pertinent if a (digital) tool is to be introduced. Where deployment occurs with community involvement, then infrastructure and policy issues will be identified more quickly than in projects that are not locally driven. Once tools work locally, it is possible that conservation organizations can consider scaling-up (see Box 3), while continuing to collaborate with communities and maintaining an awareness of how differences in local context may affect the project as it develops (Brooks et al. 2012). In short, development and deployment of a (digital) tool should take a bottom-up approach in order to ensure that while meeting the aims of the project the tool is designed for, it is also useful to those who will engage with it on the ground.

### Shifting the problem (of e-waste)

As has been shown in previous sections, digital tools are sometimes introduced to tackle very specific problems in human development. In doing so, it is possible to lose sight of the broader issues at hand and to create something that does not address the root of the problem (Alzouma 2005; Kvasny and Keil 2006). This is not to say that all technological tools designed for specific situations are inappropriate, but that in some instances it is the symptom rather than the cause that is targeted. For example, digital tools can carry a social significance, which sometimes leads to them being used more as status symbols, rather than fostering human development (Musa et al. 2005). While the introduction of such technologies may hold the promise of combatting societal problems, the reality can be that the problem is not fully addressed, or that entirely new ones are created.

There is a similar irony in conservation that in addressing specific environmental issues projects are diverting or creating problems elsewhere. There is also a danger that digital tools designed for conservation could be used against it, e.g., the potential for geo-tagged tourist photos to be used in poaching.^6^ It has to be considered that when using a digital tool, there are additional environmental and societal costs (over the use of a non-digital tool—Sui and Rejeski 2002). The use of digital tools contributes to a market in which the creation and disposal of such products has ramifications for localities (Nathan and Sarkar 2011). Many electronic devices are built with planned obsolescence—the idea that a product will only have a pre-determined operational period (Bulow 1986). Consequently, their short lifespans and inevitable disposal (Osibanjo and Nnorom 2007) have resulted in the global problem of e-waste (Fuchs 2008). Planned obsolescence, together with individual desires to own the latest gadget, means that much hardware has a shorter lifespan than it could have, resulting in high levels of electronic waste.

Electronic waste (e-waste) comprises “all types of electrical and electronic equipment (EEE) that has or could enter the waste stream” (StEP Initiative 2015). Such waste has an impact on the environment and human health, both in terms of its production and its disposal (Berkhout and Hertin 2004). It was estimated that 48.9 million metric tons of e-waste were generated globally in 2012 (StEP Initiative 2015). This amount is projected to rise to 65.4 million tons a year by 2017. The disposal of e-waste is currently regulated under the Basel Convention 2011, which monitors the global movement and disposal of hazardous wastes.

The convention was adopted in 1989, following concerns over the transboundary movement of toxic materials, particularly to less economically developed countries where there was little or no regulation of waste disposal in place. Despite the convention, it is anticipated that 50–80 % of e-waste produced is prospectively exported to developing countries (Huang et al. 2014). When not appropriately handled or contained, such waste can contaminate local waterways (Huang et al. 2014) and enter the food chain (Robinson 2009). Some of the materials ending up in the wider environment include heavy materials such as mercury and cadmium. In the US, e-waste is responsible for 70 % of heavy materials entering landfill (Widmer et al. 2005)—exposure to these materials can cause allergic reactions, brain damage, and cancer (Puckett et al. 2002), affecting both human and the natural environment.

Although reuse and recycling of e-waste is a buoyant industry in developing countries, the global movement of e-waste has ramifications for local communities (Osibanjo and Nnorom 2007; Robinson 2009). Addressing such issues requires a global effort (Nnorom and Osibanjo 2008), with large changes in practice among the industries involved (see Box 4).

**Box 4:** Case Study 4: Phonebloks—inspiring a movement, not a solution

Phonebloks is an initiative that aims “to end or reduce the various ethical and environmental problems existing in the consumer electronic market today” (https://phonebloks.com/en). It began with an idea from design student Dave Hakkens to reduce planned obsolescence in electronic products. He constructed the concept of a modular phone where each component could be replaced, rather than the entire unit. The idea caught the attention of thousands of people online and quickly gained popularity
However, Hakkens has not gone on to develop the product; instead, he has established a community of individuals—a movement—inspired toward change. This consumer pressure has stimulated a number of large companies to begin bringing the concept design into reality, such as Google’s Project Ara, which is piloting a marketable version of a modular phone
Hakkens has not shifted the problem of electronic waste by introducing a new company or product, but has instead asked an industry to confront the problems it creates

Under the ‘ecological modernisation’ movement, it was believed that digital technology would offer an opportunity to reduce environmental stress, rather than contribute to it (Jokinen et al. 1998; Murphy and Gouldson 2000). The position of conservation projects and the development of digital tools need to be more seriously considered within an international context. For instance, localized Western projects that engage citizen scientists through digital tools should be acutely aware of the broader global impact that the creation or discard of such tools has, as they may affect conservation aims directly or indirectly. If wider conservation goals can be considered alongside local aims when developing technological tools, then it could be possible to avoid addressing a conservation issue in one region while negatively contributing to another elsewhere.

## Further considerations on the (offline) impact of using digital tools

It is clear from the examples cited that, although the use of digital technology in human development can be contentious and problematic, there are very successful initiatives, which serve both the project and the people they are designed for. One of the key unifying factors of such successful projects is that they have a tangible link between the online and the offline, often through education or knowledge exchange (e.g., projects such as *Keepod*—which runs an operating system from a USB drive, allowing users to access information on any computer^7^, or *e*-*limu*—a digital tablet developed in Kenya to improve the quality of education and citizenship^8^). While projects may have set out to address a specific problem, e.g., the lack of modularity in digital products (Box 4), or a lack of communication channels (Boxes 1 and 2), they have also provided an opportunity for a local approach and ownership of the problem.

As in human development, digital technology holds the same promise to act as a valuable tool in conservation. It offers the potential to increase engagement with conservation efforts (Sandbrook et al. 2014; Arts et al. 2015b; Van der Wal et al. 2015), to improve information sharing (Banks and Burge 2004) and increase the use of local and scientific knowledge in environmental decision making (Reed 2008). However, there is also a danger that digital platforms further distance the individual from the natural world (Sandbrook et al. 2014; Verma et al. 2015). This may also be true when digital tools are used to facilitate the collection of environmental data (Maffey 2014). Furthermore, the introduction of new technologies can lead to concerns of trust in relation to who has ownership of the data and what it is being used for (Lawrence and van Turnhout 2010; Maffey et al. 2013; Arts et al. 2015b). Such limitations can be largely overcome by ensuring that digital tools are not introduced in isolation by an organization, but developed and deployed with community involvement throughout the process.

Digital technologies are increasingly used in conservation as tools to engage public communities in ‘citizen science’ projects (Newman et al. 2012). It should be considered, however, that there can be a difference between how volunteers engage with such platforms. Some can be very active ‘expert’ users, while others can be more passive. All users on the spectrum of involvement can be valuable in terms of achieving wider conservation goals. Indeed, ultimately, numbers of individuals subscribed do not equate to numbers of engaged individuals or impact on the ground. When considering the potential impact online that using a digital tool may have, it is just as important to consider the impact offline.

## Conclusion

In this Perspective, we have demonstrated that there are many similarities between the challenges faced and opportunities provided by the use of digital tools in human development and conservation. Both human development and conservation are human enterprises, and although the focus of each dimension may differ, both will benefit from a human-centric approach to technological development. From the topics covered and the cases cited within them, we suggest five key lessons that the conservation domain can take away from the use of digital technology in human development:

- Do not put all your eggs in one basket as technology will become outdated; consider existing and persisting (non) digital platforms, not just the latest development, in order to improve resilience.
- Do not let the development of digital technology reinvent the wheel in conservation projects. Consider a broad range of (existing) tools that may serve wider conservation goals.
- Take a bottom-up approach. For a digital tool to work, it has to have relevance for both the project and the communities it is to be deployed in. Once the tool works locally, consider scaling-up—but maintain an awareness of differences in local context.
- Do not shift the problem. Addressing a conservation issue in one area may lead to the creation of another one elsewhere, as is the case with e-waste.
- The impact offline is just as important as the impact online. Numbers of individuals subscribed, or number of units distributed, do not equate to numbers of engaged individuals or impact on the ground.

These lessons point to the importance of a balance of ‘sustainability’ in human development, conservation, and digital technology. In an effort to help maintain such a balance, we propose a charter for future digital conservation projects. The charter builds on the ‘Donors Charter,’ which was designed for projects in human development.^9^ We recommend that the charter be used before, and throughout, project development to help reduce replication and failure of digital innovation in conservation. We hope that it will promote collaboration in the development of digital tools and ensure that digital conservation continues to play a role in serving wider conservation goals.

## Proposed ‘digital conservation charter’

*Preliminary questions* to be asked by the project team or main stakeholders in the initiative:

1. Have the community of groups or individuals identified a problem that an appropriate form of technology may be able to address?
2. Why will the initiative benefit from technological development, and who will be using and managing it?
3. Does the team/collaborators have the necessary knowledge and experience to address both the environmental and social components of the conservation issue, as well as the technological challenges that may arise?
4. Is there already an initiative or organization working to address the conservation issue? Is collaboration possible? Have initial studies been undertaken to understand the scale of the conservation issue?
5. Does a technology or initiative currently exist (possibly in a different domain) that could be used to address the problem? Could it be adapted to address the problem?
6. What are the possible risks and undesired side effects (economically, technically, socially, and culturally) of the proposed technology?

*Implementation* of the project:

7. Will the implementation be piloted on a small scale?
8. Have financial estimates for the project been made? Is there appropriate funding for both establishment and maintenance or sustainability of the project?
9. Will you be collaborating with locally based individuals and organizations to carry out your implementation? If not, why not?
10. Are you incorporating local understanding and working practices into the technological development process? How?

*Evaluation and post*-*implementation* of the initiative:

11. How will the impact of the initiative be measured—both environmentally and socially? Do you have indicators for whether the initiative was successful or not? How will the end-users/collaborators be involved in measuring the impact of the initiative?
12. How has the initiative actively contributed to local, national, or international conservation goals?
13. Does the initiative have an exit strategy and review process? That is, if a technological solution to a conservation issue has been developed, is the local community able to continue to employ the solution without external support?
14. Will the results and technological developments be openly available for other (digital) conservation organizations and individuals to access and learn from?

## Footnotes

1 We use the term conservation to refer to the preservation and protection of the natural world—a definition that is inclusive, not exclusive, of human interaction (Adams 2009, p. xiv).

2 ‘Communities’ are here understood as social units in the same geographic location, or as groups of individuals who share practices, world views, resources, beliefs, or ideals (McMillan 1996).

3 References to developed and developing countries are used in accordance with categorization under the UN M49: http://www.itu.int/en/ITU-D/Statistics/Pages/definitions/regions.aspx.

4 http://www.android.com/one/.

5 http://www.williamkamkwamba.com/.

6 http://qz.com/206069/geotagged-safari-photos-could-lead-poachers-right-to-endangered-rhinos/.

7 http://keepod.com/.

8 http://e-limu.org/.

9 http://www.donorscharter.org/.
